# Drivers of Household Food Waste Reduction Intentions: Integrating Sustainable Marketing and Food-Related Lifestyle into the Theory of Planned Behaviors—Evidence from Tunisia

**DOI:** 10.3390/foods15162880

**Published:** 2026-08-18

**Authors:** Ahmed Yangui, Emna Ouertani, Ghassen Gandouzi

**Affiliations:** 1 Agricultural Economic Laboratory (LER–LR16INRAT07), National Institute of Agronomic Research of Tunisia INRAT, University of Carthage, Tunis 1004, Tunisia; ghassenghassen145@gmail.com; 2 Agricultural Economic Laboratory (LER–LR16INRAT07), Higher School of Agriculture of Mograne (ESAM), University of Carthage, Tunis 1004, Tunisia; emna.ouertani@esamo.ucar.tn

**Keywords:** food waste, theory of planned behavior, sustainable marketing, food-related lifestyle, developing countries

## Abstract

Household food waste represents a major environmental, economic and social challenge, particularly in developing countries where empirical evidence remains limited. To address this gap, this study extends the Theory of Planned Behavior (TPB) by simultaneously incorporating sustainable marketing and food-related lifestyle, providing a more comprehensive framework for explaining consumers’ intentions to reduce household food waste in the Tunisian context. Data were collected through a survey of 919 consumers across four Tunisian cities and analyzed using a structural equation model. The results show that attitude is the strongest predictor of food waste reduction intention, while subjective norms influence intention indirectly through attitude and perceived behavioral control. Sustainable marketing positively enhances both attitude and perceived behavioral control, whereas food-related lifestyle negatively affects attitude and behavioral intentions. The findings also indicate that stronger intentions to reduce food waste could encourage more sustainable food-related lifestyles. By integrating marketing, sensory and social meal lifestyle-related determinants into the TPB framework, this study advances understanding of household food waste behavior in an under-researched developing country context and provides practical insights for designing interventions that combine awareness raising with food management practices to promote sustainable household consumption.

## 1. Introduction

Food waste (FW) has emerged as one of the most pressing global sustainability challenges, with significant environmental, economic, and social consequences. According to the Food and Agricultural Organization of the United Nations (FAO), approximately one-third of all food produced for human consumption is either lost or wasted throughout the food supply chain [1]. This substantial loss of food not only represents an inefficient use of natural, financial and human resources but also contributes to greenhouse emissions, food insecurity, and unsustainable consumption patterns. Recognizing the magnitude of this challenge, the United Nations incorporated food waste reduction into the Sustainable Development Goals (SDGs), particularly SDG 12 on responsible consumption patterns. Target 12.3 aims to halve per capita global food waste at the retail and consumer levels by 2030 while reducing food losses along production and supply chains. Despite increasing international commitment, achieving this target remains a major challenge and continues to receive considerable attention from researchers and policymakers.

Although FW has been extensively investigated in developed countries [2,3,4,5,6], substantial knowledge gaps persist in developing regions, particularly in Africa and Asia [7,8,9,10,11,12]. Consumer-level FW has often been assumed to be lower in developing countries than in middle- and higher-income nations; however, this assumption remains insufficiently supported by empirical evidence. Consequently, there is a growing need for context-specific studies that examine household FW and its underlying determinants in these regions.

The distribution of food loss and waste also differs across countries at different stages of development. In high-income countries, the largest share of FW occurs at the retail and household consumption stages. In contrast, losses in developing countries have traditionally been concentrated in upstream stages of the food supply chain, including production, post-harvesting handling, storage, and industrial processing. However, this pattern is evolving. Rapid urbanization, rising incomes, changing lifestyles, and demographic transitions are increasingly contributing to higher levels of household FW in developing countries [12]. Despite these changes, limited evidence exists on how behavioral, psychological, socio-demographic, and contextual factors shape FW practices among urban consumers in these settings. Understanding the drivers of such behavior is therefore essential for effective interventions to reduce FW.

Recent research emphasizes that food waste should not be considered a single behavior but rather the outcome of a complex set of interconnected practices influenced by individuals’ characteristics, social norms, and institutional and environmental contexts [12]. Previous studies have identified certain consumer profiles associated with higher levels of FW, including younger consumers, women, single-person households, high-income families, and households with children [13,14,15]. Other studies have highlighted key behavioral drivers of household FW, including inadequate food storage, insufficient attention to expiration dates, inefficient meal planning and preparation, and lifestyle-related consumption habits [16,17,18,19]. While considerable research has quantified the magnitude and impacts of food waste, comparatively less attention has been devoted to understanding consumers’ behavior, perceptions, knowledge, and attitudes, particularly within developing countries.

Furthermore, Spang et al. [7] argue that existing food loss and waste research has largely focused on immediate causes while paying insufficient attention to broader structural and systemic drivers. Over recent decades, developing countries have experienced profound transformation in their food systems driven by urbanization, infrastructure development, retail modernization, globalization, consolidation within the food industry, and rising prosperity [7,18,20]. These changes have substantially reshaped food access and availability, purchasing patterns, consumption behaviors, and household food management practices, creating new challenges for food waste prevention.

Against this backdrop, the present study seeks to address an important gap in the literature by examining the determinants of household food waste reduction intentions in Tunisia, a developing country context that remains underrepresented in food waste research. Specifically, the study develops and empirically tests an extended Theory of Planned Behavior framework by integrating two additional constructs—sustainable marketing and food-related lifestyle—to better explain consumers’ intentions to reduce household food waste. Although previous studies have extended the TPB by incorporating constructs such as environmental concern, moral norms, self-identity, habits and knowledge, these extensions have primarily emphasized consumers’ internal psychological dispositions, values and capabilities. In contrast, the present study incorporates sustainable marketing and food-related lifestyle to capture two complementary dimensions that remain insufficiently integrated into TPB-based food waste research: the market-related context in which sustainable consumption is encouraged and communicated, and the lifestyle-related context in which everyday food decisions and practices take place. Sustainable marketing reflects the potential influence of sustainable-oriented market practices and communication on consumers’ attitudes, whereas food-related lifestyle captures consumers’ food-related orientations and everyday practices associated with purchasing, preparation, consumption and waste management. The proposed framework therefore goes beyond the simple addition of new predictors to the TPB by integrating psychological, market-related, and lifestyle dimensions into a unified model, thereby offering a more contextualized explanation of household food waste reduction intentions.

Using survey data collected from Tunisian consumers, the study examines the direct and indirect relationships among these constructs and identifies the key psychological and contextual factors influencing FW reduction intentions. By extending the TPB in this manner and applying it to an under-researched developing country context, the study contributes to the literature on consumer FW behavior in developing countries while providing evidence-based insights for policymakers, businesses and other stakeholders to design more effective interventions promoting sustainable food consumption.

## 2. Literature Review

According to FAO [21], FW refers to the removal of food from the supply chain that is either still suitable for consumption or has become spoiled or expired, mainly due to economic behavior, poor inventory management, or negligence. Therefore, FW is most prevalent in the later stages of the supply chain, particularly at the retail and consumer level. Chalak et al. [2] reported that up to 35% of FW can be attributed to consumer behavior. Similarly, Aktas et al. [22] emphasized that FW at the consumer level carries the most significant economic, social and environmental consequences. These include the loss of added value, the missed opportunity to feed individuals experiencing hunger, and the depletion of natural resources, biodiversity, labor, and energy. However, consumers’ FW behavior is a multifaceted issue, shaped by a complex interplay of behavioral factors. The decision-making process that ultimately leads to wasting food is influenced by a range of social, economic and personal factors, and emerges from the interaction of personal values, choices, and levels of engagement [3]. Therefore, gaining a deeper understanding of the drivers of household FW is essential for designing effective policy interventions and achieving outcomes aligned with the Sustainable Development Goals [4].

Multiple studies have identified a strong conceptual connection between FW and individual food-related preferences and behaviors. Factors such as nutritional concerns, food safety awareness [23], dietary mindfulness [24], emotional attachment to food [25], and even the cultural and social value attributed to food [26] all influence household FW. Routine domestic practices and ingrained habits also play a key role [27], along with basic food preferences [28].

Moreover, consumer purchasing behaviors significantly impact how food is managed at home. Inadequate meal planning, disorganized shopping habits, and impulsive buying often lead to over-purchasing, which increases the risk of spoilage and waste [24,25,26,29,30]. Additionally, the social norm of being a ‘good provider’—ensuring abundance and variety for all household members—can further drive excessive acquisition [31,32]. Aktas et al. [22] further emphasize that seasonal dietary shifts and the generation of surplus food also contribute to higher FW levels.

Other influential drivers include external factors such as product packaging, labeling clarity, and marketing strategies [6,33]. Psychosocial dimensions—such as lifestyle choice, eating patterns, culinary competences, and personal values—also play a crucial role [34]. Sensory attributes like freshness, texture, color, smell, and food taste heavily influence food rejection [9,35]. Furthermore, labeling and esthetic standards—often perceived as indicators of quality and/or safety—can lead consumers to prematurely discard food to avoid health risks or to adhere to a preference for freshness [6,36,37].

Despite growing global interest in consumer FW, research remains limited in many regions, particularly across Africa. African countries like Tunisia have seen little investigation into household FW behaviors, leaving significant gaps in understanding the underlying drivers and dynamics of FW in these contexts [7].

Available data on FW in Tunisia is scarce. In one of the few descriptive studies, Sassi et al. [8] identified cereal-based products—particularly bread, pasta, and rice—as the most commonly wasted food items, followed by vegetables and dairy products. The study also found that Tunisian consumers tend to rely heavily on their sensory evaluation (appearance, smell, and taste) to determine food edibility. Key reasons for discarding food included prolonged storage, excessive portion sizes, and signs of spoilage. In a different context, Jribi et al. [9,10] investigated how crisis situations, such as COVID-19 pandemic, affected consumer awareness, attitudes and behavior regarding FW in Tunisia. Their findings suggest short-term stress and uncertainty, particularly fear of food shortages, heightened awareness of FW and prompted behavioral adjustments. Many consumers adopted food management practices aligned with FAO recommendations [38], such as better planning and reduced waste. These behavioral shifts were closely associated with individual knowledge levels and socio-demographic factors, including age and employment status. To sustain the positive behavioral changes driven by the pandemic, targeted education and communication strategies are essential. Campaigns should focus on deepening public understanding of FW’s broader impacts, emphasizing the benefits of prevention, and equipping households with practical tools to improve food-related decision-making.

Meanwhile, studies examining consumer-related FW in North African and Arab countries remain scarce. A limited number of studies, including Abiad et al. [39], have investigated this issue, particularly in Egypt, Algeria, and Morocco. Abdelradi [12] found that in Egypt, food waste is primarily influenced by waste management practices, food pricing, and consumer choices, with planning and shopping routines playing a central role—largely driven by advanced marketing strategies. Similarly, Arous et al. [40] observed that in Algeria, a significant portion of food waste occurs at the household level, with poor planning and shopping habits as key predictors. This behavior tends to fluctuate during specific periods, such as Ramadan, when the most wasted food categories are fruits, vegetables, cereals, and bakery products.

Belfakira et al. [41] highlighted that in Moroccan households the key reasons for discarding food items include difficulties in reusing small leftovers and limited cooking skills using available ingredients. Additionally, Zoubi et al. [42] identified social class as a major driver of FW: higher-income households tend to purchase—and waste—more food. FW also correlates with socio-demographic factors such as age, gender, and family size, with older men in large, wealthy families being more prone to waste. Notably, FW persists even among consumers who claim to be aware of the issue and report adopting responsible eating habits [42].

The context of FW generation and consumer behavior in Sub-Saharan African countries may differ significantly from that of North African nations. However, there remains a substantial lack of comprehensive data on FW across most African regions. Abu Hatab et al. [11] investigated the determinants of FW behavior among urban consumers in Ethiopia using an extended Theory of Planned Behavior (TPB) framework. Their findings identified self-identity, attitudes, and perceived behavioral control as the most significant predictors of consumers’ intention to reduce FW. While consumers may feel that reducing FW is within their control, they often perceive it as a difficult task to implement in practice. This highlights the need to consider not only psychological factors, but also consumers’ practical skills, abilities, and purchasing habits when analyzing FW behavior.

Cronjé et al. [43] identified leftover food, as well as fruits and vegetables, as the main contributors to FW in South Africa. Key drivers included bulk purchasing, buying incorrect products or quantities, and insufficient meal planning. These practices contribute to significant economic losses, with Nahman and De Lange [44] estimating that the total cost of food waste throughout South Africa’s food value chain is equivalent to approximately 2.1% of the country’s GDP. A cross-continental comparison by Peronti et al. [45], involving four regions in Europe (Germany, Italy, Poland, and Denmark) and Morocco as an African country, suggests that family size plays a key role in shaping FW behavior—larger households tend to waste less food per person than smaller ones. The study also found that rural households tend to manage food waste more effectively than those in urban areas.

To date, most African studies on FW have been exploratory in nature, focusing mainly on FW quantification, typologies, causes and consumer perceptions. Exceptions include Abu Hatab et al. [11] and Abdelradi [12], who analyzed consumer FW behavior in Egypt and Ethiopia, respectively, using structural equation modeling based on the TPB framework to identify the psychological and behavioral drivers of FW decisions.

Therefore, this study contributes to the existing literature by:

(i) Extending current knowledge on consumer FW behavior in African contexts where research remains limited.
(ii) Proposing the first conceptual model specifically designed to investigate the behavioral factors influencing household food waste (FW) behavior among Tunisian consumers.
(iii) Developing a TPB-based conceptual framework that incorporates novel variables such as food-related lifestyle and the influence of sustainable marketing.

## 3. Conceptual Model: Food Waste Reduction Intention

Drawing on an extended application of the TPB, Abu Hatab et al. [11] found that consumers’ intention to reduce food waste is primarily influenced by three factors: their self-concept as responsible individuals, their attitudes towards FW reduction, and their perceived capacity to perform such behaviors. Their findings indicate that while many consumers believe they possess the necessary ability and authority to reduce FW, translating these intentions into consistent daily practices remains challenging. This suggests the existence of an intention–behavior gap, where perceived control does not always result in effective implementation.

**Figure 1:**
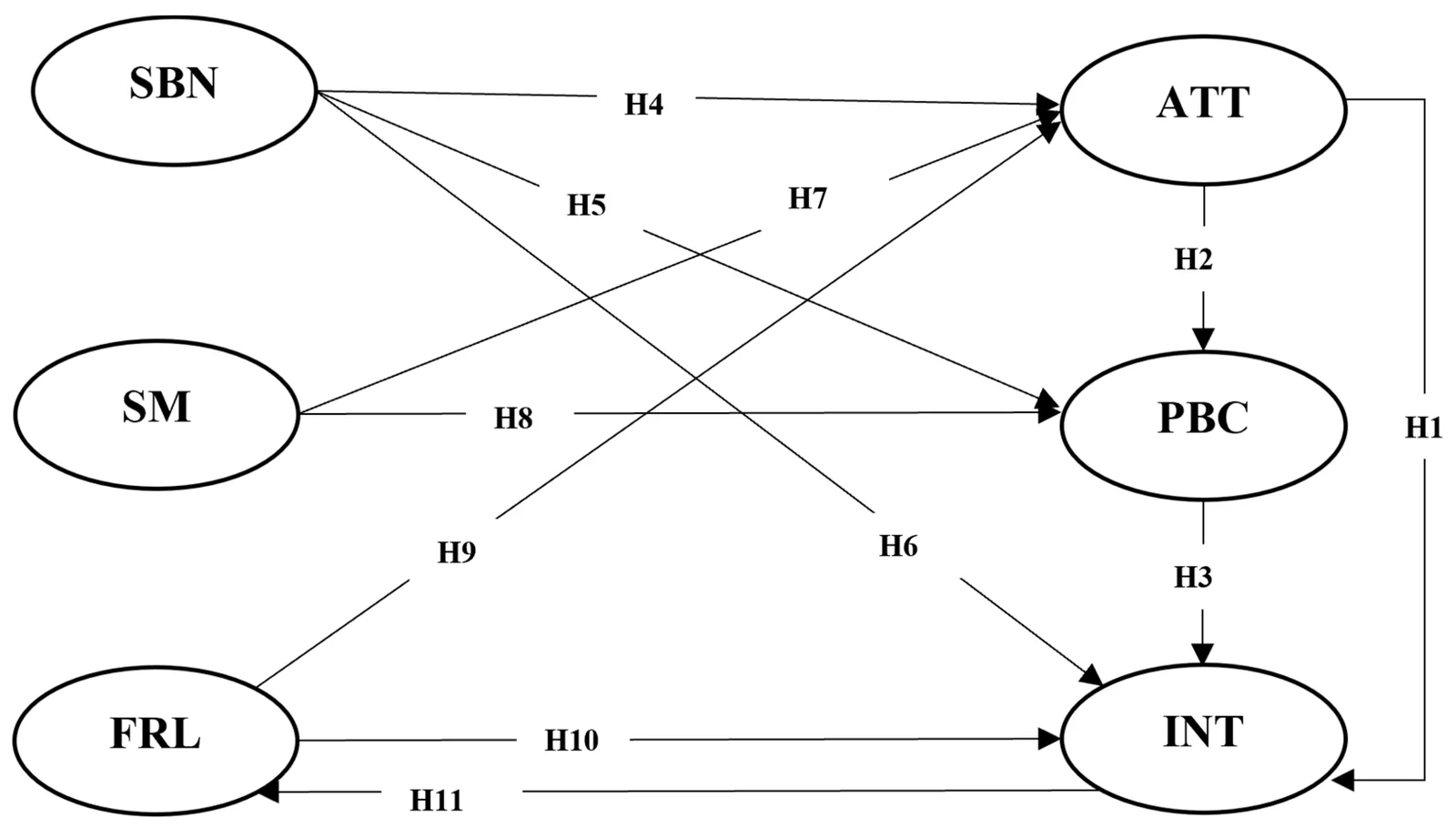
Extended TPB conceptual model regarding food waste behavior.

Figure 1 presents a proposed conceptual framework of food waste reduction intention, grounded in an extended TPB. Beyond the traditional TPB determinants, the model integrates sustainable marketing and food-related lifestyle as additional explanatory variables. The framework depicts the decision-making pathways through which these factors influence consumers’ food waste intentions, providing a comprehensive understanding of the determinants of food waste in contemporary contexts.

According to the TPB, attitude is a key determinant of behavioral intention. Individuals who view food waste reduction as beneficial, important, and worthwhile are expected to be more motivated to adopt behaviors aimed at minimizing food waste.

**H1.**  *Consumers with more favorable attitudes (ATT) towards food waste reduction are more likely to exhibit stronger intentions (INT) to reduce food waste.*

**H2.**  *Individuals who have a favorable attitude toward a behavior may be more motivated to acquire knowledge, skills and experience, which can increase their perceived ability to perform the behavior.*

Perceived behavioral control (PBC) reflects individuals’ perceptions of their ability to perform a particular behavior. In the context of food waste reduction, consumers who believe they have the necessary resources, knowledge, skills, and opportunities to prevent food waste are more likely to develop stronger intentions to engage in food-saving practices.

**H3.**  *Consumers who perceive that they possess the necessary resources, knowledge, skills, and opportunities to prevent food waste are more likely to develop stronger intentions to reduce it.*

The TPB also emphasizes the role of subjective norms (SBN), which refer to perceived social pressure from significant others. In the context of food waste reduction, individuals who believe that people important to them who approve minimizing food waste are more likely to develop stronger intentions to engage in food-saving behaviors [14,22,46]. Therefore, social expectations may play a significant role in shaping consumers’ intentions to reduce food waste.

Additionally, social influence may not only shape consumers’ intentions but also affect their attitudes and perceived behavioral control. Previous studies suggest that support and encouragement from family members, peers, and society can enhance individuals’ confidence and perceived capability to perform a behavior [47,48]. Therefore, subjective norms are expected to positively influence consumers’ attitudes towards food waste reduction, strengthen their perceived behavioral control, and increase their intention to minimize food waste.

**H4.**  *Consumers are more likely to develop positive attitudes towards food waste reduction when they perceive that important referent groups expect and encourage them to engage in food waste reduction behaviors.*

**H5.**  *Subjective norms positively influence consumers’ perceived behavioral control regarding food waste reduction.*

**H6.**  *Subjective norms positively influence consumers’ intention to reduce food waste.*

### 3.1. Sustainable Marketing (SM)

In addition to psychological determinants, marketing strategies adopted by grocery retailers may also influence consumers’ food waste behavior. Previous research has shown that marketing stimuli, such as promotional offers, product display, and sales incentives, can alter consumers’ purchase habits and contribute to food waste generation [3,29]. Conversely, sustainable marketing practices—including social marketing campaigns, sustainability-focused advertising, eco-labeling, awareness campaigns, retailer communication, and digital marketing initiatives—can encourage more responsible consumption patterns and contribute to food waste reduction [49,50,51]. Research by Chinie et al. [51] and Aschemann-Witzel et al. [52] further suggests that marketing communications, particularly those implemented at the point of sale, can educate consumers about the consequences of food waste and promote behavioral change by influencing consumers’ perceptions, awareness, and decision-making processes. Consequently, sustainable marketing is expected to enhance consumers’ attitude towards food waste reduction and strengthen their perceived behavioral control.

**H7.**  *Sustainable marketing positively influences consumers’ attitudes toward food waste reduction*.

**H8.**  *Sustainable marketing positively influences consumers’ perceived behavioral control regarding food waste reduction*.

### 3.2. Food-Related Lifestyle (FRL)

Although direct empirical tests of the effect of food involvement and food-related lifestyle on attitude toward food waste remain scarce, there is a well-established theoretical relationship between food-related lifestyle and consumer’ attitudes towards food waste in the literature [53,54]. In general, consumers with a higher food-related lifestyle develop more negative attitudes toward food waste because they attach greater value to food, possess better food-related knowledge and skills, and engage more actively in food management. Consequently, they tend to perceive food waste as undesirable and are more willing to adopt food waste prevention behaviors [30,55,56,57]. However, this relationship may be moderated by contextual factors such as household characteristics, convenience orientation, income, and social norms [3,12,52].

**H9.**  *Food-related lifestyle significantly influences consumers’ attitudes toward food waste reduction.*

Furthermore, people who value food experiences, enjoy food, invest time in food and attach social importance to meals often develop a greater appreciation of food; consequently, they may be less willing to waste it and more motivated to reduce food waste. Consumers who attach greater social and sensory value to food are expected to develop stronger intentions to reduce food waste because they place higher value on food and the experience surrounding its consumption [30,58]. The curiosity and interest in food may foster a greater appreciation of food resources, leading them to be more conscious of waste prevention and more willing to engage in waste reduction practices.

**H10.**  *Food-related lifestyle positively influences consumers’ intention to reduce food waste.*

The TPB posits that behavioral intention is the immediate antecedent of actual behavior [59]. Since consumers are generally averse to waste [60], intentions to avoid wasting food are expected to influence their actual food-related lifestyle. Once these intentions are formed, consumers may adjust their food-related practices by limiting activities that could lead to excess food, thereby reducing the intensity of social and sensory food involvement. Food waste reduction intentions can lead people to change their food-related practices, becoming more restrained, mindful, and efficiency-oriented [46,61].

**H11.**  *Consumers’ intention to reduce food waste could have a positive effect on their food-related behavior.*

It is important to note that the original FRL instrument encompasses several dimensions that capture how individuals integrate food into their daily lives [62,63,64]. However, subsequent research has frequently adopted adapted or shortened versions of the FRL construct by selecting dimensions that are theoretically relevant to the specific research context while preserving its underlying lifestyle perspective [63]. Consistent with this approach, FRL does not necessarily need to include all possible food practices; it can be adapted according to the behavioral context investigated [62,64]. Therefore, the present study focused exclusively on the social and sensory dimensions of food consumption, as they were considered particularly relevant to understanding household food-related behaviors associated with food waste. This adaptation was guided by the study objectives and the need to maintain a concise questionnaire, thereby reducing respondent burden and fatigue during face-to-face interviews while preserving satisfactory measurement reliability and validity [65]. Accordingly, the findings should be interpreted as reflecting the influence of these specific dimensions rather than the complete multidimensional FRL construct.

Furthermore, the relationship between FRL and food waste reduction intention is specified as reciprocal in the present study. The rationale for this specification is that FRL may influence consumers’ evaluations of food and food waste and consequently shape their intention to reduce food waste, while stronger food waste reduction intentions may, in turn, be associated with greater attention to food-related practices and potentially reinforce particular aspects of consumers’ food-related lifestyle. The reciprocal specification therefore reflects a theoretically plausible mutual relationship rather than an assumption of unidirectional causality. However, this relationship should be interpreted as structural associations rather than causal effect. The reciprocal paths are estimated within the complete structural system rather than as an isolated two-variable feedback loop. The model incorporates exclusion restrictions through the broader network of relationships among subjective norm, sustainable marketing, attitude, perceived behavioral control, food-related lifestyle, and intention (Figure 1).

## 4. Materials and Methods

### 4.1. The Sample

To achieve the study objectives and test the proposed conceptual model, a household survey was conducted in 2022 across the four governorates of Greater Tunis, namely Tunis, Ben Arous, Manouba and Ariana, which collectively account for approximately one-quarter of Tunisia’s total population and therefore provide a relevant setting for examining household food-related behaviors and food waste reduction intentions. The target population comprised individuals aged 18 years or older who were fully or partially responsible for food purchasing within their household. Given the absence of a comprehensive sampling frame identifying individuals according to their responsibility for household food purchasing, a non-probability convenience sampling approach was adopted. Participants were recruited through face-to-face interviews at different locations across the four governorates, including supermarkets, public transportation areas, universities, and other public locations. The questionnaire began with a screening question to verify participants’ eligibility; individuals younger than 18 years or those were not fully or partially responsible for household food purchasing were excluded from the survey. Participation was voluntary, and respondents were not required to provide personally identifying information and were informed about the purpose of the study before completing the questionnaire. Following data collection and screening, 919 valid questionnaires were retained for the final analysis. Given the non-probability nature of the sampling procedure, the sample cannot be considered statistically representative of the Tunisian population. Table 1 presents the socio-demographic characteristics of the respondents, including gender, age, area of residence, educational attainment, income, household composition, and the presence of dependent children.

### 4.2. Measures

The questionnaire consisted of three main sections and began with a screening question to determine whether the respondent was fully or partially responsible for food purchasing within the household. Only eligible participants were invited to complete the survey.

The first section collected respondents’ socio-demographic information, including gender, age, education, income, household composition, and other relevant characteristics. The second section focused on participants’ food purchasing, consumption, and food management practices. The third section was designed to measure the constructs included in the conceptual model. Most of these constructs were assessed using five-point Likert scales ranging from strong disagreement to strong agreement.

The questionnaire was administrated through face-to-face interviews in the metropolitan areas of the four governorates comprising Greater Tunis. On average, each interview lasted between 15 and 20 min, although the duration varied depending on respondents’ characteristics, such as age, familiarity with the topic of FW, and the pace of the interview.

### 4.3. SEM Model Specification

To examine the proposed conceptual model for assessing consumers’ intentions to reduce food waste, structural equation modeling (SEM) was employed. SEM is a well-established multivariate statistical technique widely used in consumer behavior research because it enables the simultaneous estimation of multiple interrelated dependence relationships involving both observed and latent variables [66]. Unlike conventional regression methods, SEM allows the inclusion of latent constructs—such as attitudes, behavioral intentions, subjective norms, and food-related lifestyle—which cannot be directly observed but are inferred from multiple measured indicators.

According to Jöreskov and Sörbomm [67], SEM comprises two main components: the measurement model and the structural mode. The measurement model specifies the relationships between latent constructs and their observed indicators, whereas the structural model describes the causal relationships among the latent constructs. The identification of latent variables requires the specification of multiple observed indicators, typically represented by responses to several items in a stated-preference questionnaire. These relationships are evaluated through Confirmatory Factor Analysis (CFA), which assesses the validity and reliability of the latent constructs.

The measurement model for the exogenous latent variables is expressed as:

(1) *x* = ***Λx***
***ξ*** + ***δ***

where *x* is a q × 1 vector of observed exogenous variable, ***Λx*** is a q × n matrix of factor loading relating the observed variables to the exogenous latent variables, ***ξ*** is an n × 1 vector of latent exogenous constructs, and ***δ*** is a q × 1 vector of measurement error. The measurement errors are assumed to be uncorrelated with the latent variables.

Similarly, the measurement model for the endogenous latent variables is defined as:

(2) *y* = ***Λy***
***η*** + ***ε***

where *y* is a *p* × 1 vector of observed endogenous indicators, ***Λy*** is a *p* × m matrix of factor loading, ***η*** is an m × 1 vector of endogenous latent constructs, and ***ε*** is a *p* × 1 vector of measurement errors. It is assumed that the error terms are uncorrelated with the endogenous latent variables.

The structural component of the SEM specifies the hypothesized causal relationships among the latent constructs, both exogenous and endogenous constructs, and is represented as:

(3) *η* = *β*
***η*** + ***Γ***
***ξ*** + ***ζ***

where *β* is an m × m matrix representing the relationships among endogenous latent variables (***η***), ***Γ*** is an m × n matrix of structural coefficients describing the effects of exogenous latent variables (***ξ***) on endogenous latent variables (***η***), and ***ζ*** is a m × 1 vector of structural disturbances representing unexplained variations.

The adequacy of the proposed SEM was evaluated using several complementary criteria. First, the statistical significance of the estimated parameters was examined to assess the strength of the hypothesized relationships. Second, the direction and magnitude of the estimated coefficients were evaluated for consistency with underlying theoretical framework. Finally, the overall model fit was assessed using a range of commonly recommended goodness-of-fit indices [68,69,70,71,72,73]. These included the Chi-square ($\chi_{2}$) statistic and the root-mean-square error of approximation (RMSEA) and standardized root mean residual (SRMR) as measures of absolute model fit values, together with the Goodness-of-Fit Index (GFI), Adjusted Goodness-of-Fit Index (AGFI), Comparative Fit Index (CFI), Normed Fit Index (NFI), Non-Normed Fit Index (NNFI) (also known as the Tucker–Lewis Index, TLI) and Incremental Fit Index (IFI) as incremental measures. Collectively these indices provide a comprehensive assessment of the extent to which the proposed model adequately reproduces the observed covariance structure.

## 5. Results and Discussion

### 5.1. Sample Descriptive Results

The first section of the results presents the descriptive statistics of the study sample. Table 1 summarizes the socio-demographic characteristics of the 919 participants. The sample was evenly distributed by gender, suggesting that responsibility for household food purchasing is generally shared between men and woman. Since participation was restricted to individuals involved in household food purchasing decisions, the sample provides insights into the characteristics of consumers who actively participate in food acquisition and management practices. Regarding age distribution, the majority of participants were younger than 40 years of age, indicating a relatively young sample profile. All marital status categories were represented, although most respondents were either single or married. While this composition reflects the participation of diverse household profiles, the relatively high proportion of younger and single respondents should be considered when interpreting the findings, as household structure and life stage may influence food purchasing routines and food waste behaviors.

Regarding educational attainment, the distribution of participants across low, medium and high education levels was relatively balanced, indicating a heterogeneous sample in terms of educational background. The average household size was 4.75 members, suggesting that most participants lived in households comprising four to five individuals.

In terms of household composition, 15% of participants reported having at least one child younger than six years of age, while 28% reported having at least one child younger than twelve years. These characteristics are relevant because household size, the presence of children, and family organization may influence food purchasing patterns, meal planning and food waste generation. With respect to household income, more than half of the participants belonged to the low- and middle-income categories, with monthly household incomes not exceeding 2000 Tunisian dinars (TND).

Overall, the sample presents heterogeneity in terms of socio-demographic characteristics, although the relatively young age profile and proportion of single respondents should be taken into account when considering the generalizability of the results to the broader Tunisian population.

### 5.2. Latent Variables and Indicators Description

The proposed conceptual model examining consumers’ intentions to reduce food waste comprises six latent constructs. Four constructs—attitude, perceived behavior control, subjective norm and consumers’ behavioral intentions—represent the core components of TPB [59]. To capture the influence of sustainability-oriented factors, the model extends the original TPB by incorporating two additional constructs: sustainable marketing, reflecting consumers’ perception of retailers’ sustainability initiatives, and food-related lifestyle, representing consumers’ eating habits and the role of food in their daily lives and overall well-being.

Table 2 presents the measurement indicators for each latent construct, together with the corresponding mean scores and standard deviation (SD). Overall, respondents reported favorable attitudes toward food waste reduction and indicated that they were raised in environments where wasting food was discouraged. They also perceived that family members and close social circles shared similar values and encouraged practices aimed at minimizing food waste, resulting in relatively high subjective norm scores. Likewise, respondents expressed strong intentions to adopt food waste reduction practices within their households.

In contrast, perceived behavioral control received comparatively a considerable rating, suggesting that although respondents recognize the importance of reducing food waste, they encounter difficulties in consistently implementing waste reduction practices. These challenges appear to be particularly associated with food storage and maintaining appropriate conditions for preserving food.

The descriptive statistics also reveal generally positive perceptions of sustainable marketing and food-related lifestyle. Mean scores for sustainable marketing ranged from 3.855 to 4.220, indicating broad support for sustainable marketing initiatives, particularly the use of information and communications technologies (ICT) to raise awareness of food waste, the integration of QR codes communicating companies’ food waste reduction strategies, and enhanced food labeling providing information on nutritional value, food waste, and environmental impacts. For food-related lifestyle, respondents moderately agreed that eating is a multisensory experience (score = 3.960) and that meals and food preparation constitute important aspects of their social life (score = 3.853).

Overall, mean scores above the scale midpoint indicate positive attitudes toward food waste reduction, favorable social norms, strong behavioral intentions, and positive perceptions of sustainable marketing and food-related lifestyle. Standard deviation below 1.10 indicated moderate response variability and a satisfactory level of consensus across these constructs. By comparison, perceived behavioral control exhibited lower mean score (3.231–3.470) and greater variability, suggesting that respondents differ more substantially in their perceived ability to effectively implement food waste reduction behaviors.

### 5.3. Measurement Model Results

Item reliability, convergent and discriminant validity

The results presented in Table 3 demonstrate that all latent constructs achieved composite reliability (CR) values exceeding 0.80, thereby indicating a high level of internal consistency reliability and confirming that the measurement items consistently capture their respective constructs [74].

The convergent validity of the measurement model, which reflects the extent to which indicators of the same constructs share a high proportion of common variance, was subsequently assessed using indicator outer loading and the average variance extracted (AVE). Initially, the standardized outer loadings of all indicators were examined. The majority of the indicators exhibited outer loading values above the recommended threshold of 0.70, indicating satisfactory indicator reliability and demonstrating that the latent constructs account for a substantial proportion of the variance in their respective indicators [70]. Indicators with loadings above this threshold imply that approximately 50% or more of the variance in the observed measures is explained by the underlying latent constructs. Furthermore, all constructs reported AVE values greater than the recommended minimum threshold of 0.50, indicating that each construct explains more than half of the variance of its indicators. These findings provide strong evidence of adequate convergent validity of the measurement model [74].

The discriminant validity of the measurement model was evaluated using both the indicator cross-loadings and the Fornell–Larcker criterion. Examination of the cross-loadings confirmed that each indicator loaded more strongly on its corresponding latent construct than on any other construct, demonstrating that the indicators adequately represent their intended constructs. Subsequently, the Fornell–Larcker criterion was applied by comparing the square root of the AVE for each construct (displayed in bold along the diagonal of Table 4) with the inter-construct correlation (off-diagonal elements). As presented in Table 4, the square root of the AVE for every latent construct exceeded its highest correlation with any other construct. Moreover, all inter-construct correlation coefficients remained below the recommended threshold 0.85, further supporting the distinctiveness of the constructs. Collectively, these results confirm that each latent variable shares greater variance with its own indicators than with other latent variables, thereby providing robust evidence of discriminant validity [74].

### 5.4. Confirmatory Factor Analysis (CFA) Measurement

To validate the measurement model, a Confirmatory Factor Analysis (CFA) was conducted for the six latent constructs prior to estimating the structural model (Table 3). CFA is recommended to assess the adequacy of the measurement model and to identity potential specification problems, such as excessively high correlations among constructs, large standard errors, or poorly performing indicators [70]. The overall results indicate that the proposed measurement model exhibits an acceptable fit to the observed data, thereby providing a sound basis for subsequent structural equation modeling.

As presented in Table 3, all goodness-of-fit indices satisfy the recommended threshold values reported in the literature [69,70]. The Satorra–Bentler scaled Chi-square statistic was 170.455 with 57 degrees of freedom (df). The corresponding normed Chi-square (NC = $\chi_{2}$/df) was 2.99, which falls within the commonly accepted range of 1 to 5, indicating an acceptable model fit.

The absolute fit indices further support the adequacy of the measurement model. Specifically, the Root Mean Square Error of Approximation (RMSEA) and the Standardized Root Mean Square Residual (SRMR) were 0.0466 and 0.0346, respectively. Both values are well below the recommended cutoff of 0.08, indicating a close fit between the hypothesized measurement model and the observed covariance matrix. The incremental fit indices also provide strong evidence of model adequacy. The Comparative Fit Index (CFI) reached 0.985, substantially exceeding the recommended threshold of 0.90. Similarly, the Normed Fit Index (NFI), Non-Normed Fir Index (NNFI/TLI), Goodness-of-Fit Index (GFI), and Incremental Fit Index (IFI) all exceeded 0.90, further confirming the satisfactory fits of the measurement model.

Overall, the CFA results demonstrate that the proposed measurement model possesses adequate psychometric properties and provides a satisfactory representation of the observed data. Consequently, the measurement model was considered valid and reliable, justifying the estimation and evaluation of the structural modem in the subsequent stage of the analysis.

### 5.5. Extended Theory of Planned Behavioral Model Results

The results indicated that the SEM provided a good fit to the data. As reported in Table 5, the incremental fit indices were all above the recommended threshold of 0.95, with NFI = 0.974, CFI = 0.982, IFI = 0.982 and NNFI = 0.970. Furthermore, the model demonstrated excellent absolute fit, as evidenced by RMSEA and SRMR values of 0.0493 and 0.039, respectively, both falling below the recommended cutoff of 0.05. These results provide strong evidence of the model’s overall goodness of fit.

TPB basic determinants of food waste reduction intentions

The SEM results presented in Table 5 indicate that consumers’ positive attitudes towards food waste reduction exert a significant and positive influence on their intention to reduce household food waste (β = 0.970, *p* < 0.01), thereby supporting hypothesis H1. According to TPB, attitude is one of the primary determinants of behavioral intention, suggesting that individuals who evaluate a behavior positively are more likely to develop a strong intention to perform it. The present result is also in agreement with recent empirical studies demonstrating that favorable attitudes toward food conservation significantly enhance consumers’ intentions to minimize food waste [54,58,75].

The strong positive relationship observed in this study suggests that consumers who perceive food waste as morally unacceptable, environmentally harmful, and economically inefficient are more likely to adopt intentions aimed at reducing food waste. Such attitudes often reflect deeply rooted personal values and social norms acquired through family upbringing, cultural traditions, and ethical beliefs that emphasize respect for food as a valuable and limited resource. Moreover, positive attitudes towards food waste reduction are frequently reinforced by awareness of the broader societal consequences of food waste. Consumers increasingly recognize that discarding edible food is ethically problematic in a world where millions of people continue to experience food insecurity and hunger [30,76].

An unexpected finding of the present study is the absence of a significant direct relationship between subjective norms and consumers’ intention to reduce household food waste (H6: β = 0.054, *p* > 0.10). This result suggests that perceived social pressure from family members, friends, or other significant referents does not directly motivate Tunisian consumers to adopt food waste reduction intentions. According to the TPB [59], subjective norms reflect individuals’ perceptions of whether important others expect them to perform a particular behavior. Consequently, previous research has generally argued that stronger normative expectations lead to stronger behavioral intentions [54,58]. However, the findings of the present study do not support this proposition in the context of household food waste reduction.

The lack of a significant direct effect is nevertheless consistent with several empirical studies conducted in different cultural contexts. For example, Cammarelle et al. [77] and Chun T’ing et al. [78] similarly reported that subjective norms were not significant predictors of food waste reduction intentions. Rather than exerting a direct influence, normative beliefs appeared to operate indirectly by shaping consumers’ attitudes and their perceived behavioral control. The present findings provide further support for this interpretation, as subjective norms significantly and positively influenced both attitudes (H4: β = 0.239, *p* < 0.01) and perceived behavioral control (H5: β = 0.182, *p* < 0.01). These results suggest that social influence contributes to the formation of favorable evaluations of food waste reduction and enhances consumers’ confidence in their ability to prevent food waste, even though it does not directly translate into behavioral intentions.

One possible explanation for this pattern lies in the social and cultural characteristics of the Tunisian context. Food purchasing, preparation, and consumption are generally regarded as private household decisions, limiting the extent to which individuals perceive external expectations regarding food waste management. In other words, the decision to minimize food waste appears to be driven more by internalized beliefs than by perceived social approval or disapproval. This interpretation may be particularly relevant during culturally significant occasions such as Ramadan, a period characterized by profound religious and social values but also by substantial changes in food consumption patterns. Social norms during Ramadan may inadvertently normalize food surplus rather than encourage food waste prevention.

According to TPB, perceived behavioral control constitutes the third determinant of behavioral intention and refers to an individual’s perception of the ease or difficulty associated with performing a given behavior [59]. This construct reflects the extent to which individuals believe they possess the necessary resources, opportunities, and capabilities to successfully execute the target behavior. Within the context of food waste reduction, previous research has consistently identified perceived behavioral control as a significant predictor of both behavioral intentions and actual food waste-related behaviors [29,78,79]. Individuals who perceive greater control over their ability to prevent or reduce food waste are generally more likely to develop stronger intentions to engage in food waste reduction practices, as they view such behaviors as feasible and manageable [58]. Conversely, lower levels of perceived behavioral control are expected to weakens individuals’ confidence in their capacity to perform food waste reduction behaviors, thereby diminishing their intention to engage in such practices.

However, the empirical findings of the present study do not support this theoretical proposition by providing for hypothesis 3 a significant positive path coefficient (H3: β = 0.349, *p* < 0.01). This finding suggests that, despite perceiving various barriers to reducing food waste, consumers remain motivated to engage in waste reduction practices. Such barriers may include limited knowledge of proper food storage techniques, insufficient time for meal planning, uncertainty regarding the interpretation of food date labels, or household-related constraints that reduce individuals’ perceived capacity to effectively manage food. Nevertheless, these perceived obstacles do not completely undermine their intention to reduce food waste. Individuals may recognize the importance of reducing food waste because of environmental, ethical, or economic concerns, even when they doubt their ability to consistently perform the required behaviors. Consequently, perceived behavioral control continues to explain variations in intention—as reflected by the significant positive path coefficient—while other motivational factors such as favorable attitudes towards food waste reduction may sustain relatively high levels of intention despite lower perceived control.

Furthermore, an interesting finding is that attitudes have a significant negative effect on perceived behavioral control (H2: β = −0.209, *p* < 0.01). Consumers may hold very positive attitudes toward reducing food waste because they recognize its environmental, economic, and ethical importance. However, this heightened awareness may simultaneously make them more conscious of the practical challenges involved. In other words, the more they value food waste reduction, the more they recognize how difficult it is to consistently achieve it. Consequently, positive attitudes do not necessarily translate into greater perceived behavioral control. A consumer strongly believes that reducing food waste is important; at the same time, they often buy food impulsively, they have little time to plan meals, and family members have unpredictable eating habits. In this study, perceived behavioral control was operationalized through consumers’ perceived ability to manage food preservation and storage practices and was assessed through negatively worded items, where higher values indicate greater perceived constraints and lower control over food waste reduction behaviors. Therefore, the negative association between attitudes towards reducing food waste could be perceived with fewer barriers and difficulties in implementing waste reduction practices, reflecting a positive effect of attitudes on perceived behavioral control. Accordingly, although the estimated path coefficient is negative due to the reverse direction of the PBC measurement scale, its theoretical interpretation supports the expected positive relationship between attitudes and perceived behavioral control. Therefore, hypothesis H2 was considered supported.

#### 5.5.1. Sustainable Marketing

Consistent with previous research, the present study demonstrates that sustainable marketing is significantly and positively associated with both consumers’ attitude toward food waste reduction and their perceived behavioral control. Specifically, sustainable marketing exerts a positive and significant effect on consumers’ attitudes toward food waste reduction (H7: β = 0.246, *p* < 0.05), indicating that exposure to sustainability-oriented marketing initiatives fosters more favorable evaluations of food waste reduction behaviors. This finding suggests that marketing strategies emphasizing the environmental, economic, and social consequences of food waste can strengthen consumers’ awareness of the benefits associated with waste prevention, thereby encouraging a more positive disposition toward adopting food waste reduction practices. This result is consistent with previous studies showing that social marketing campaigns, sustainability-focused advertising, eco-labeling, and digital communications initiatives effectively promote pro-environmental attitudes and responsible consumption behaviors [50,51,52].

Moreover, sustainable marketing positively influences consumers’ perceived behavioral control regarding food waste reduction (H8: β = 0.273, *p* < 0.01). This finding suggests that sustainable marketing not only motivates consumers by developing favorable attitudes but also augments their confidence in their ability to reduce food waste. This finding is consistent with Soma et al. [50], who demonstrated that consumer awareness interventions can influence food waste-related behaviors by improving knowledge, awareness, and engagement with waste reduction practices. Therefore, sustainable marketing should not be considered only as a persuasive communication strategy but also as a mechanism that provides consumers with practical resources and behavioral capabilities. In this sense, through educational messages, practical guidance, and behavioral recommendations—including advice on meal planning, proper food storage, portion management, and the interpretation of food date labels—sustainable marketing can equip consumers with knowledge and skills necessary to perform food waste reduction behaviors more effectively. By reducing uncertainty and increasing consumers’ perceived competence, these marketing initiatives strengthen individuals’ perceptions that food waste reduction is feasible and within their control.

Chinie et al. [51] highlighted that awareness campaigns can contribute to combating food wasting behavior by increasing consumers’ understanding of the consequences of food waste and encouraging more responsible practices. However, the present study extends this perspective by showing that sustainable marketing influences food waste reduction intentions through both attitudinal and perceived control mechanisms.

Collectively, these findings suggest that sustainable marketing contributes to food waste reduction through two complementary psychological pathways. First, it supports consumers’ positive evaluations of food waste reduction by reinforcing its environmental, economic and societal value. Second, sustainable marketing fortifies consumers’ perceived capability to implement food waste reduction practices by providing actionable knowledge and reducing perceived behavioral barriers. These dual effects highlight the strategic role of sustainable marketing as both a persuasive and an empowering mechanism for promoting sustainable consumption behaviors.

#### 5.5.2. Food-Related Lifestyle

The present study also investigated the influence of food-related lifestyle (FRL) on consumers’ attitude and behavioral intentions toward reducing household food waste. The results indicate that FRL has a significant negative effect on both consumers’ attitudes (H9: β = −0.653, *p* < 0.01) and their behavioral intention to minimize household food waste (H10: β = −0.466, *p* < 0.01). These findings suggest that the FRL characteristics exhibited by the respondents are associated with less positive evaluations of food waste prevention and weaker intentions to engage in waste reduction behaviors. It is important to note that, in the present research, the FRL construct was not intended to capture the entire spectrum of the food-related lifestyle dimensions. Instead, an adapted operationalization was adopted, focusing specifically on the sensory and social aspects of meals, which reflect consumers’ preferences, experiences and attitudes toward meal enjoyment, social interaction, and eating practices. The present study emphasizes the role of sensory and social meal-related characteristics as potential drivers of food waste prevention attitudes and intentions. Therefore, the observed relationship between FRL and food waste-related outcomes should be interpreted within the specific conceptual boundaries of the FRL dimensions examined in this research.

The findings appear to differ from the general conclusion that consumers with higher food-related lifestyle develop more negative attitudes toward food waste because they attach greater value to food, possess better food-related knowledge and skills, and engage more actively in food management. Consequently, they tend to perceive food waste as undesirable and are more willing to adopt food waste prevention behaviors [30,55,56,57]. However, recent studies suggest that certain modern FRLs—particularly those characterized by convenience orientation, frequent dining with friends or family members, impulsive purchasing, excessive food variety seeking, and preference for abundance—can inadequately increase household food waste. Accordingly, hypothesis H10 was not considered supported with respect to the hypothesized positive direction, but the relationship itself is statistically significant and occurs in the opposite direction. For example, Stancu et al. [30] found that poor meal planning and spontaneous shopping significantly increase household food waste because consumers tend to over-purchase food and underestimate future consumption. Similarly, van Geffen et al. [6] reported that modern lifestyles characterized by busy schedules and convenience-oriented food practices reduce consumers’ ability to effectively manage food, resulting in greater food waste. These studies suggest that not all food-related lifestyle dimensions encourage sustainable food management. More recent studies also support this interpretation. Boulet et al. [80] argued that consumers with convenience-driven lifestyles often prioritize time-saving and flexibility over careful food management, increasing the likelihood of food spoilage and disposal. Likewise, Schanes et al. [81] observed that households with highly dynamic lifestyles frequently experience mismatches between food purchasing and actual consumption, leading to unnecessary waste despite awareness of food waste issues.

The strong negative relationship found in this study may therefore indicate that the respondents’ food-related lifestyle reflects behaviors such as limited meal planning, impulsive buying, preference for fresh and abundant food, and lower engagement in food management practices. These lifestyle characteristics may weaken positive attitudes toward food waste reduction because consumers perceive food waste as an unavoidable consequence of maintaining convenience and food quality. Consequently, these attitudes translate into weaker behavioral intentions to reduce household food waste.

Another possible explanation is offered by Aschemann-Witzel et al. [52], who argued that food-related lifestyle influences food waste indirectly through everyday routines and habitual consumption patterns rather than through environmental concern alone. Even consumers who express concern about sustainability may generate substantial food waste when their lifestyle emphasizes convenience, variety, or frequent purchasing. This perspective helps explain why the current study found a negative association between food-related lifestyle and food waste reduction intentions. In particular, lifestyles that prioritize convenience and flexibility often lead to less structured meal planning, more frequent shopping trips, and a tendency to over-purchase perishable items. Similarly, a preference for dietary variety or fresh products can increase the likelihood of food spoilage before consumption, especially when households lack consistent strategies for storage or leftover management. Furthermore, consumers who highly value the sensory aspects of food may exhibit stronger preferences for freshly prepared meals and premium-quality ingredients while showing less willingness to consume leftovers or foods perceived as having diminished freshness. Research has consistently shown that esthetic expectations and concerns about food quality are among the main drivers of avoidable household food waste, as consumers frequently discard food that remains edible but no longer meets their desired sensory standards [6,52]. These habitual behaviors operate largely at an automatic or routine level, meaning that, for example, pro-environmental attitudes alone are insufficient to prevent waste generation.

Overall, these findings suggest that promoting sustainable household food management requires more than increasing awareness of food waste. Interventions should encourage practical lifestyle changes such as meal planning, shopping with a list, portion management, proper food storage, and creative use of leftovers. Such strategies may help transform food-related lifestyles into patterns that support positive attitudes and stronger intentions to reduce household food waste [46].

The findings further suggest that consumers’ behavioral intention to reduce food waste has the potential to reshape their food-related lifestyle by encouraging more deliberate and sustainable food management practices (H11: β = 0.998, *p* < 0.01). This result is consistent with the argument that intentions to reduce food waste are not merely psychological dispositions but can translate into tangible changes in everyday food-related behaviors and shape the way consumers experience and organize their eating habits. As consumers become more committed to minimizing food waste, they are more likely to adopt practices such as meal planning, purchasing only the quantities needed, making better use of leftovers, and update their limit of sensory standards. These behaviors reflect a transition toward a more mindful food-related lifestyle.

Consumers with stronger intentions to reduce food waste may become more conscious of balancing enjoyment of food with responsible consumption. Rather than abandoning the social and pleasurable aspect of eating, they may adopt more thoughtful practices during meal preparation and food consumption, such as preparing appropriate portion sizes for social gatherings, making better use of leftovers, and selecting food quantities that satisfy both expectations and sustainability goals. In this way, food waste reduction intentions encourage a more mindful approach to food experiences without necessarily diminishing the enjoyment associated with eating. This is consistent with Barone et al. [46], who argue that intentions to reduce food waste encourage consumers to reconsider their everyday food practices, promoting greater restraint and awareness in food purchasing, preparation, and consumption. Similarly, Giannou and Mantzios [61] suggest that food waste reduction intentions can gradually reshape consumers’ food-related practices by fostering more reflective and efficient habits. As these intentions become integrated into daily routines, they may influence not only food management behaviors but also the broader meaning attached to food, including how meals are shared and enjoyed.

Generally, the findings suggest that intentions to reduce food waste can promote a transformation in consumers’ food-related lifestyle by encouraging them to integrate sustainability into their social and sensory experiences of food. Rather than reducing the pleasure associated with eating, stronger intentions may foster a more balanced lifestyle in which food enjoyment coexists with more efficient and responsible consumption practices.

## 6. Conclusions

The present research work extends the TPB by integrating sustainable marketing and food-related lifestyle to explain household food waste reduction intentions in the Tunisian context. By proposing and empirically validating an integrated framework that combines psychological, marketing, and lifestyle-related factors, this study provides new insights into the mechanisms underlying consumers’ engagement in food waste reduction practices, particularly in an emerging economy context. The findings confirm that attitude is the strongest predictor of consumers’ intention to reduce food waste, while perceived behavioral control also exerts a positive influence. Environmental, ethical, cultural and economic concerns appear to sustain consumers’ intentions, indicating that favorable attitudes can compensate, to some extent, for lower perceived behavioral control. In contrast, subjective norms do not directly affect intention, but are indirectly associated with behavioral intention by strengthening attitudes and perceived behavioral control, suggesting the predominance of internalized beliefs over social pressure.

The study further demonstrates that sustainable marketing is indirectly associated with food waste reduction by simultaneously developing more favorable attitudes and supporting consumers’ confidence in their ability to adopt waste prevention practices. Conversely, food-related lifestyle emerges as a significant barrier, as convenience-oriented and less structured food management routines weaken both attitudes and intentions to reduce food waste. At the same time, stronger intentions to reduce food waste positively influence food-related lifestyle, suggesting a reciprocal process through which sustainable intentions can gradually reshape everyday consumption habits.

Overall, these findings provide both theoretical and practical contributions by demonstrating that food waste reduction intentions cannot be fully explained by traditional TPB determinants alone and require the consideration of broader contextual and behavioral factors. This study contributes to the literature by showing how sustainable marketing and food-related lifestyle complement the TPB framework in explaining household food waste reduction behavior. From a practical perspective, the findings emphasize that effective food waste reduction strategies should combine awareness-building with practical intervention aimed at improving consumers’ food planning and food management skills and encouraging more sustainable lifestyle practices. Such integrated approaches can support the transition toward more responsible household food consumption and contribute to broader sustainability goals.

Despite its contributions, this study presents several limitations that should be considered when interpreting the findings. First, the study relies on self-reported responses, which may be subject to social desirability. Respondents may have overreported socially desirables attitudes or behaviors, particularly regarding food waste reduction, potentially leading to discrepancies between reported and actual behaviors. Second, the empirical investigation was conducted among household food purchasers in the Greater Tunis metropolitan area. Although this area represents an important share of the Tunisian population and provides a relevant context for studying household food behaviors, the findings should not be generalized to other Tunisian regions. Regional differences in income levels, lifestyles, food cultures, and access to food markets may influence food waste-related attitudes and intentions. Third, in the present study, FRL is measured using only two indicators reflecting sensory and social meal-related characteristics. Although these indicators capture dimensions of FRL that are theoretically relevant to consumers’ perceptions and intentions regarding food waste, FRL is a broad and multidimensional construct that may also encompass other dimensions, such as planning meals, price consciousness, food preparation practices, and purchasing routines. The findings should therefore be interpreted specifically in relation to the sensory and social dimensions captured by the selected indicators rather than being generalized to FRL as a whole. Future research should employ more comprehensive and multidimensional measures of FRL, allowing researchers to examine whether different food-related lifestyle dimensions exert distinct or even opposite effects on food waste attitudes and reduction intentions. Finally, although the study provides new insights by integrating psychological, marketing and lifestyle-related factors within an extended TPB framework, future research could further enrich the model by incorporating additional determinants of food waste behaviors, such as hospitality norms, shopping routines, and situational factors like religious and cultural events.

## Figures and Tables

**Table 1:** Sample socio-demographic description.

Factors	Levels	Frequency	% of the Sample
Gender			
	Man	467	50.82
	Woman	452	49.18
Age			
	From 18 to 29 years	450	48.97
	From 30 to 39 years	204	22.20
	From 40 to 49 years	141	15.34
	From 50 to 59 years	95	10.34
	More than 60 years	26	2.83
Education Level			
	Illiterate	67	7.29
	Primary studies	252	27.42
	Secondary studies	128	13.93
	Professional studies	119	12.95
	Graduate studies	353	38.41
Civil status			
	Single	474	51.58
	Married	377	41.02
	Divorced	34	3.70
	Widowed	20	2.18
	Other	14	1.52
Zone of residence			
	South	70	7.62
	Center	723	78.67
	North	126	13.71
Family composition			
	Average	4.74	
	1 < *p* < 6 years	138	15.02
	7 < *p* < 12 years	128	13.93
	13 < *p* < 18 years	353	38.41
	19 < *p* < 55 years	894	97.28
	*p* > 55 years	525	57.13
Working status			
	Worker	127	13.82
	Retired	29	3.16
	Employee	155	16.87
	Senior manager	119	12.95
	Craftsman/tradesman/entrepreneur	129	14.04
	farmer	43	4.68
	Unemployed	145	15.78
	Student	171	18.61
Income/month			
	Less than 500 TND	55	5.98
	From 501 to 1000 TND	114	12.40
	From 1001 to 1500 TND	177	19.26
	From 1501 to 2000 TND	161	17.52
	From 2001 to 2500 TND	163	17.74
	From 2501 to 3000 TND	97	10.55
	From 3001 to 3500 TND	83	9.03
	More than 3500 TND	69	7.51

*p*: indicates the presence of persons within the specified age interval in the household; TND: Tunisian dinar.

**Table 2:** Latent variables and indicators description.

Factors and Indicators	Means	SD
Attitude (ATT)		
ATT1: I was raised with the belief that food should not be wasted.	3.942	1.039
ATT2: I believe that food should not be wasted.	4.083	0.844
Perceived Behavioral Control (PBC)		
BC1: I find it difficult to store food under the required conditions.	3.231	1.260
BC2: I sometimes find it difficult to store certain types of food products.	3.394	1.353
BC3: I have difficulty storing food at high temperatures (e.g., summer season).	3.470	1.295
Subjective Norm (SBN)		
SBN1: The people close to me have special practices to reduce food waste.	3.198	1.278
SBN2: Most of the people who matter to me think that reducing food waste is highly desirable.	3.700	1.160
Intention (INT)		
INT1: I intend not to throw away any food.	3.986	0.993
INT2: I intend to find a new use for leftover food.	3.769	1.044
Sustainable Marketing (SM)		
SM1: I think it is time to revise packaging labels by providing information about consumers’ actual nutritional needs, the quantities wasted, the negative effects on the environment, etc.).	3.911	1.088
SM2: This is important if, in addition to the use-by date, we add to the label another date after which the product retains its maximum nutritional quality.	3.981	1.092
SM3: It is very interesting for me to find a “QR code” link that presents the company’s strategy to combat food waste.	3.855	1.078
SM4: It is very interesting to use ICT to raise awareness about food waste and to propose solutions.	4.22	0.892
Food-Related Lifestyle (FRL)		
FRL1: Meals and food preparation are an important part of my social life.	3.853	1.022
FRL2: Eating for me is about touching, smelling, tasting and seeing; all the senses are involved, it is a very exciting sensation.	3.960	1.002

**Table 3:** Confirmatory Factor Analysis (CFA) results and reliability measurements.

Factors	Outer Loading Standardized	Average Variance Extracted (AVE)	Composite Reliability (CR)
Attitude (ATT)		0.683	0.809
ATT1	0.721		
ATT2	0.920		
Perceived Behavioral Control (PBC)		0.693	0.871
PBC1	0.799		
PBC2	0.898		
PBC3	0.798		
Subjective Norm (SBN)		0.696	0.815
SBN1	0.655		
SBN2	0.982		
Intention (INT)		0.718	0.835
INT1	0.894		
INT2	0.798		
Sustainable marketing (SM)		0.669	0.889
SM1	0.821		
SM2	0.823		
SM3	0.854		
SM4	0.772		
Food-related lifestyle (FRL)		0.671	0.796
FRL1	0.628		
FRL2	0.974		
Goodness-of-fit	Chi-square = 170.455; df = 57; NC = 2.99 < 3; RMSEA = 0.0466 < 0.08; *p*-value of close fit (RMSEA < 0.05) =0.748; NFI = 0.978 > 0.95; CFI = 0.985 > 0.95; IFI = 0.985 > 0.95; NNFI = 0.973 > 0.95; SRMR = 0.0346 < 0.05; GFI = 0.954 > 0.9.		

**Table 4:** Fornell–Larcker test of discriminant validity.

Latent Variables	Attitudes	Subjective Norms	Perceived Behavioral Control	Intention	Sustainable Marketing	Food-Related Lifestyle
Attitudes	**0.826**					
Subjective norms	0.171	**0.834**				
Perceived behavioral control	−0.252	0.160	**0.832**			
Intention	0.463	0.216	0.077	**0.847**		
Sustainable marketing	0.133	0.091	0.265	0.157	**0.836**	
Food-related lifestyle	0.012	0.129	0.243	0.210	0.200	**0.819**

The bold values along the diagonal of the Table represent the square root of the AVE for each construct.

**Table 5:** Structural equation model results.

Hypothesis	R^2^	Path Coefficients	Standard Errors	*t*-Value	Status
Attitude (ATT)	29.8%				
H4: SBN → ATT		0.239 ***	0.047	3.802	Accepted
H7: SM → ATT		0.246 **	0.042	4.182	Accepted
H9: FRL → ATT		−0.653 ***	0.208	3.601	Accepted
Perceived behavioral control (PBC)	18.3%				
H2: ATT → PBC		−0.209 ***	0.208	3.016	Accepted
H5: SBN → PBC		0.182 ***	0.063	3.494	Accepted
H8: SM → PBC		0.273 ***	0.040	6.580	Accepted
Intention (INT)	22.2%				
H1: ATT → INT		0.970 ***	0.230	5.220	Accepted
H3: PBC → INT		0.349 ***	0.088	4.444	Accepted
H6: SBN → INT		0.054	0.084	0.873	Rejected
H10: FRL → INT		−0.466 ***	0.198	3.337	Rejected
Food-related lifestyle (FRL)	54.4%				
H11: INT → FRL		0.998 ***	0.172	4.126	Accepted
Goodness-of-fit	Chi-square = 206.785; df = 64; NC = 3.23 < 5; RMSEA = 0.0493 < 0.08; *p*-value of close fit (RMSEA < 0.05) = 0.547; NFI = 0.974 > 0.95; CFI = 0.982 > 0.95; IFI = 0.982 > 0.95; NNFI = 0.970 > 0.95; SRMR = 0.039 < 0.05; GFI = 0.944 > 0.9.				

***, ** and * indicate that the corresponding parameter is statistically significant at the 1%, 5% or 10% level, respectively.

## Data Availability

The original contributions presented in the study are included in the article; further inquiries can be directed to the corresponding author.

## Abbreviations

The following abbreviations are used in this manuscript:

TPB	Theory of Planned Behavior
SEM	structural equation model
CFA	Confirmatory Factor Analysis
FW	food waste
ATT	attitude
PBC	perceived behavioral control
SBN	subjective norm
INT	intention
SM	sustainable marketing
FRL	food-related lifestyle
RMSEA	Root Mean Square Error of Approximation
SRMR	Standardized Root Mean Residual
GFI	Goodness-of-Fit Index
AGFI	Adjusted Goodness-of-Fit Index
CFI	Comparative Fit Index
NFI	Normed Fit Index
NNFI	Non-Normed Fit Index
TLI	Tucker–Lewis Index
IFI	Incremental Fit Index
